# Chordin-like 1 is a novel prognostic biomarker and correlative with immune cell infiltration in lung adenocarcinoma

**DOI:** 10.18632/aging.203814

**Published:** 2022-01-12

**Authors:** Bing Deng, Xiaorui Chen, Lingfang Xu, Li Zheng, Xiaoqian Zhu, Junwei Shi, Lei Yang, Dian Wang, Depeng Jiang

**Affiliations:** 1 Department of Respiratory Medicine, The Second Affiliated Hospital of Chongqing Medical University, Chongqing, China

**Keywords:** lung adenocarcinoma, CHRDL1, chordin-like 1, prognosis, TCGA

## Abstract

Chordin-like 1 (CHRDL1), an inhibitor of bone morphogenetic proteins(BMPs), has been recently reported to participate in the progression of numerous tumors, however, its role in lung adenocarcinoma (LUAD) remains unclear. Our study aimed to demonstrate relationship between CHRDL1 and LUAD based on data from The Cancer Genome Atlas (TCGA). Among them, CHRDL1 expression revealed promising power for distinguishing LUAD tissues form normal sample. Low CHRDL1 was correlated with poor clinicopathologic features, including high T stage (OR=0.45, *P*<0.001), high N stage (OR=0.57, *P*<0.003), bad treatment effect (OR=0.64, *P*=0.047), positive tumor status (OR=0.63, *P*=0.018), and TP53 mutation (OR=0.49, *P*<0.001). The survival curve illustrated that low CHRDL1 was significantly correlative with a poor overall survival (HR=0.60, *P*<0.001). At multivariate Cox regression analysis, CHRDL1 remained independently correlative with overall survival. GSEA identified that the CHRDL1 expression was related to cell cycle and immunoregulation. Immune infiltration analysis suggested that CHRDL1 was significantly correlative with 7 kinds of immune cells. Immunohistochemical validation showed that CHRDL1 was abnormally elevated and negatively correlated with Th2 cells in LUAD tissues. In conclusion, CHRDL1 might become a novel prognostic biomarker and therapy target in LUAD. Moreover, CHRDL1 may improve the effectiveness of immunotherapy by regulating immune infiltration.

## INTRODUCTION

Lung cancer remains the malignant tumor with the highest mortality around the world and has seriously burdened our health and economics for a long time [1, 2]. Non-small cell lung cancer (NSCLC) is the largest category of lung cancer and accounts for 80%-85% of the total lung cancer. Among them, lung adenocarcinoma (LUAD) is the most common subtype of NSCLC [3]. Patients with early LUAD do not have specific symptoms, so they often progress to advanced patients at the time of diagnosis. There are many therapy methods for advanced patients in LUAD, including chemotherapy, radiotherapy, targeted therapy and immunotherapy [4]. In the past ten years, targeted therapy has made significant progress in lung cancer [5]. Several genes have been applied as drug targets, such as epidermal growth factor receptor (EGFR), anaplastic lymphoma kinase (ALK), ROS1, RET, HER2, Kras and MET [6–8]. Drugs explored based on these genes have shown exciting efficacy [9]. However, due to the high heterogeneity, complexity and progression of cancer, the available biological targets are not satisfactory [10]. Thus, it is vital to continually identify more efficient prognostic biomarkers and other potential therapy targets [11].

CHRDL1 (Chordin-like 1) is a kind of secretory protein belonging to the Chordin family [12]. Its function is similar to that of Chordin, mainly as a specific inhibitor of bone morphogenetic proteins (BMPs) [13]. The initial study suggested that CHRDL1 played an essential role in anterior segment development and cortical neuronal development [14, 15]. Recently, researchers found that CHRDL1 could participate in the progression of several tumors, such as malignant melanoma, leukemia, breast cancer and gastric cancer [16–19]. Moreover, the prognostic value of CHRDL1 has also been mentioned recently. In breast cancer and thyroid cancer, patients with high CHRDL1 had a poorer prognosis [20, 21]. However, its role of CHRDL1 in LUAD remains unclear.

Our study performed the differential expression of CHRDL1 between LUAD patients and normal samples based on the TCGA database. The association between CHRDL1 and clinicopathologic features was evaluated, as well as the prognostic significance. Then it was grouped according to its expression level. And Function of clustering and enrichment of pathways of CHRDL1 were used to expound the underlying mechanism in LUAD by GO, KEGG and GSEA analysis. Moreover, we comprehensively discuss the possible influence of CHRDL1 on immunotherapy through analyzing the relevance between CHRDL1 and immune infiltration. Finally, experimental verification identified the specific expression of CHRDL1 and favorable prognostic value in LUAD.

## MATERIALS AND METHODS

### RNA-sequencing profiles containing clinic data from TCGA and GTEx data repository

A total of 513 cases of gene expression profile (HTSeq-FPKM and HTSeq-counts) containing clinical data from LUAD projects were collected from TCGA. Whether it contained clinical data as the exclusion criteria in this study. And transcripts per million reads (TPM) transformed from level 3 HTSeq-FPKM profiles were used for the subsequent analyses. Unavailable or unknown clinical characteristics in 513 patients were considered as missing values [22]. All the displayed analysis adopted the value of log_2_ (TPM+1). The operating guidelines stated by TCGA (https://www.cancer.gov/tcga) were strictly implemented. The RNAseq data for differential expression and pan-cancer analysis were obtained from UCSC XENA (https://xenabrowser.net/datapages/). The Toil process was regarded as a unified processing program to obtain TCGA and GTEx data in TPM format [23].

### Screening of significant DEGs (differential expression genes) based on CHRDL1 in LUAD

DESeq2(3.8) package was applied to screen the significant DEGs based on CHRDL1 expression in LUAD [24]. The threshold values for the DEGs were set as |log2(Fold Change)|>1.5 and adjusted P value<0.05.

### GO and KEGG enrichment analysis

ClusterProfiler(3.6.0) software was implemented to perform GO function and KEGG pathway enrichment analysis on the DEGs in high/low CHRDL1 expression group respectively [25].

### Gene set enrichment analysis (GSEA)

In the study, GSEA, conducted by R package clusterProfiler(3.6.0), was used to illuminate the differences of functional clustering and enrichment pathways between high and low CHRDL1 groups [26]. The gene set permutations in each analysis were set as 1000 times. A phenotype label was generated according to the expression level of CHRDL1. And the pathways enrichment of MSigDB Collection (c2.cp.v7.0.symbols.gmt) were performed based on adjusted P value<0.05 and FDR q-value<0.25.

### The correlation between CHRDL1 expression and immune infiltration

Single-sample gene set enrichment analysis (ssGSEA) method was applied for the immune infiltration analysis in LUAD. And the GSVA package of R(3.6.0) was used to calculated for 24 types of immune cells in tumors, including B cells, T cells, CD8 T cells, natural killer (NK) cells [27], CD56bright natural killer cells (NK CD56bright cells), CD56dim natural killer cells (NK CD56dim cells), regulatory T cells (Treg), central memory T cells (Tcm), effector memory T cells (Tem), gamma delta T cells (Tgd), T follicular helper (Tfh), dendritic cells (DCs), immature dendritic cells (iDCs), activated dendritic cells (aDCs), plasmacytoid dendritic cells (pDCs), mast cells [28], neutrophils, eosinophils, macrophages, cytotoxic cells, T helper cells, type-1 T helper cells (Th1), type-2 T helper cells (Th2), and type-17 helper cells (Th17) [29, 30]. According to the signature genes of 24 types immunocytes in the literature [31], the gene expression data of each tumor sample was used to quantify the relative enrichment score of every immunocyte. Spearman correlation was used to analyze the relationship between CHRDL1 and these immune cells, and the differences of immune cell infiltration between the high and low CHRDL1 expression groups were analyzed by Wilcoxon rank sum test.

### Establishment of protein-protein interaction (PPI) network

PPI network of DEGs based on CHRDL1 expression was established by String online database (http://string-db.org), and the filter criteria was the interaction with combined>0.4 [32]. Cytoscape(3.7.2) software was applied to visualize the PPI network in this study.

### Immunohistochemistry (IHC) experiment reagents and clinical samples

A total of 204 clinical samples were collected from the Department of Respiratory Medicine, The Second Affiliated Hospital of Chongqing Medical University. They contain 102 LUAD tissues and 102 normal samples. Ethics approval of the Hospital Institutional Board and informed consent from the patients were obtained(Chongqing, China). Clinical tissue samples were made into paraffin sections in pathology department. Dewaxing of paraffin sections was performed in oven at 56° C for 2 hours. Antigen retrieval and blocking were applied by citrate buffer and 3% H_2_O_2_. Then incubation of the primary antibody was applied on sections overnight at 4° C by anti-CHRDL1 (GTX117884, GeneTex, USA) and anti-CD30 (GTX01872, GeneTex, USA). On the second day, the sections were continually incubated at 37° C for 1 hour with HRP-conjugated secondary antibody (G1213, Servicebio, China). Moreover, staining counterstain of the sections were worked by DAB coloration kits (G1212, Servicebio, China) and hematoxylin (G1004, Servicebio, China). Dehydration of the sections was applied with a graded series of ethanol. Finally, Image-Pro Plus software was used to quantify the IHC staining intensity of the sections by integrated optical density (IOD).

### Statistical analysis

The statistical analysis and plots were performed using R(v.3.6.0). Receiver operating characteristic (ROC) analysis, Wilcoxon rank sum test and signed rank test were implemented in the expression of CHRDL1 in normal samples and tumor samples [33, 34]. Kruskal-Wallis and Wilcoxon rank sum test were performed to assess relationships between clinicopathologic features and the specific expression of CHRDL1. The Pearson X^2^ test, t test and logistic regression were applied to analyze the correlation between clinicopathologic variables and low/high CHRDL1 expression group. Survival curve based on Kaplan-Meier method were drawn to assess the prognostic value of CHRDL1 on overall survival (OS), disease-specific survival (DSS) and progression-free survival (PFS). Cox logistic regression models were constructed to identify independent variables by univariate and multivariate regression analysis [35]. And then, the rms (https://cran.r-project.org/web/package/rms/index.html) packages was applied to establish a nomogram with these independent variables. The concordance index (C-index) was calculated to evaluate the predictive ability of the model. A calibration curve was constructed to evaluate the prediction accuracy of the nomogram based on the prognostic model [36].

## RESULTS

### Expression differences of CHRDL1 in tumor tissues and normal samples

**Figure 1:**
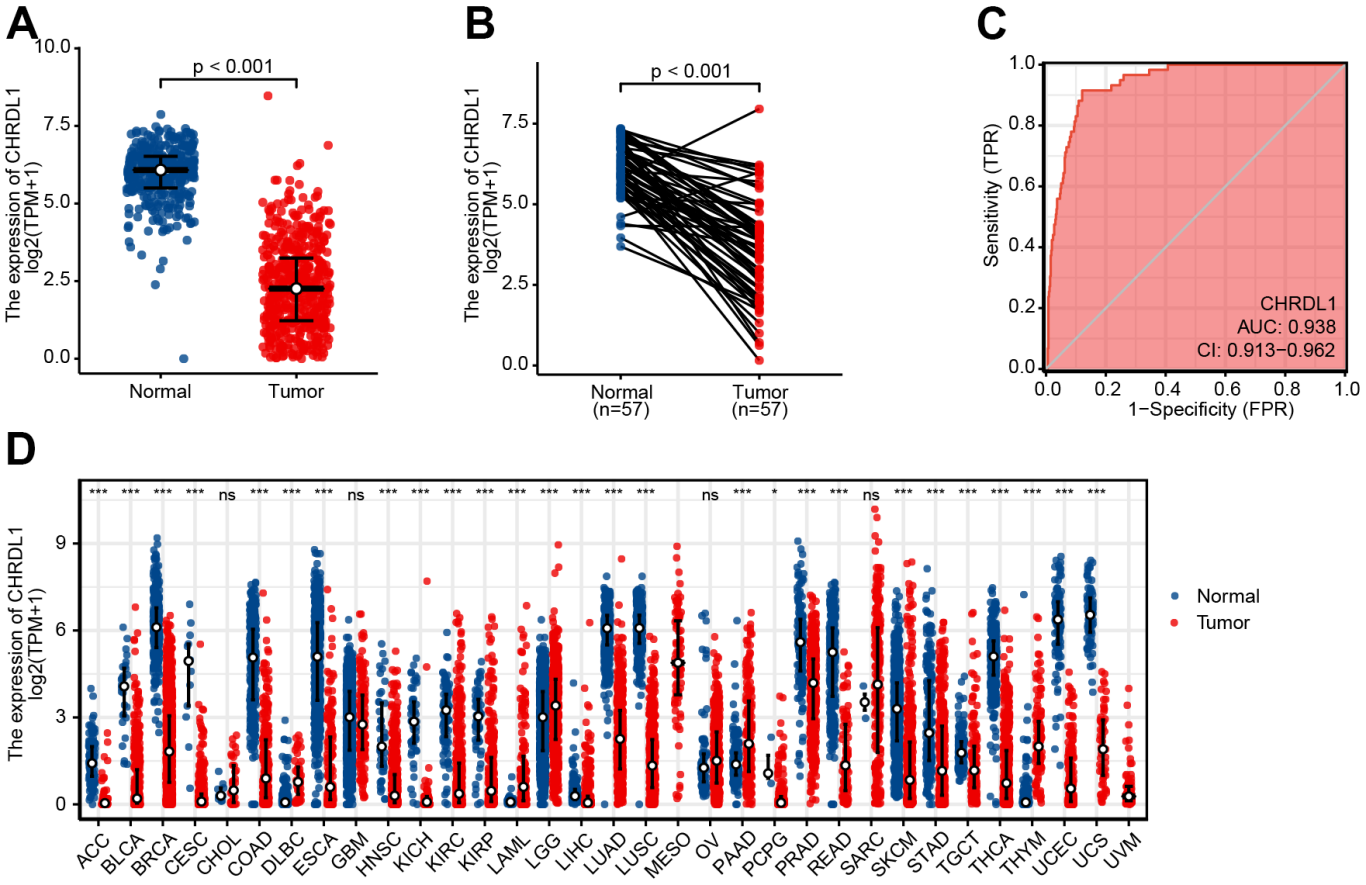
**Expression difference of CHRDL1 in normal samples and tumour samples.** (**A**) Expression of CHRDL1 in normal samples from TCGA and GTEx and tumour sample of LUAD. (**B**) Expression levels of CHRDL1 in paired tumour and adjacent samples of LUAD. (**C**) ROC analysis of CHRDL1 expression showing promising discrimination power between tumor and non-tumor tissues in LUAD. (**D**) Expression levels of CHRDL1 in Pan-cancer. ns, P{greater than or equal to}0.05; *, P<0.05; **, P<0.01; ***, P<0.001.

The expression of CHRDL1 was visualized in normal samples and LUAD samples based on GTEx and TCGA. Obviously, CHRDL1 expression of tumor tissues was significantly decreased compared with normal samples in LUAD (Figure 1A, *P*<0.001). And the consistent results were found in paired tumor tissues and adjacent samples from TCGA (Figure 1B, *P*<0.001). Moreover, CHRDL1 expression revealed promising discrimination power as the ROC curve showed that the AUC of CHRDL1 expression for identifying LUAD from normal tissues was 0.938(CI=0.913-0.962, Figure 1C). Pan-cancer analysis showed CHRDL1 was also low expressed in most types of tumors (Figure 1D).

### CHRDL1 expression correlated with clinicopathologic features

The clinicopathologic features of LUAD patients were downloaded from TCGA, including gender, race, TNM stage, pathologic stage, primary therapy outcome, smoking history, TP53 status and KARS status (Table 1). The results illustrated that CHRDL1 was significantly correlative with T stage(*P*<0.001), N stage(*P*=0.008), pathologic stage(*P*=0.004), tumor status(*P*=0.023), TP53 status(*P*<0.001), gender(*P*=0.003) and age(*P*=0.003). And there was no relation between CHRDL1 expression and other clinicopathologic features [37].

**Table 1:** Association between CHRDL1 expression and clinicopathologic features based on TCGA.

**Characters**	**Level**	**Expression of CHRDL1**		***P value***
		**Low**	**High**	
n		257	256	
T stage	T1	62(24.2%)	106(41.7%)	<0.001
	T2	154(60.2%)	122(48.0%)	
	T3	27(10.5%)	20(7.9%)	
	T4	13(5.1%)	6(2.4%)	
N stage	N0	153(59.8%)	177(72.2%)	0.008
	N1	60(23.4%)	35(14.3%)	
	N2	41(16.0%)	33(13.5%)	
	N3	2(0.8%)	0(0.0%)	
M stage	M0	176(93.1%)	168(93.3%)	1.000
	M1	13(6.9%)	12(6.7%)	
Pathologic stage	Stage I	118(46.5%)	156(62.2%)	0.004
	Stage II	73(28.7%)	48(19.1%)	
	Stage III	50(19.7%)	34(13.5%)	
	Stage IV	13(5.1%)	13(5.2%)	
Primary therapy outcome	CR	147(69.7%)	168(78.1%)	0.127
	PD	39(18.5%)	29(13.5%)	
	PR	2(0.9%)	4(1.9%)	
	SD	23(10.9%)	14(6.5%)	
Gender	Female	121(47.1%)	155(60.5%)	0.003
	Male	136(52.9%)	101(39.5%)	
Race	Asian	5(2.3%)	2(0.9%)	0.435
	Black or African American	27(12.3%)	25(11.0%)	
	White	187(85.4%)	200(88.1%)	
Smoker	No	30(12.0%)	44(17.7%)	0.090
	Yes	221(88.0%)	204(82.3%)	
Tumor status	Tumor free	128(57.4%)	160(68.1%)	0.023
	With tumor	95(42.6%)	75(31.9%)	
TP53 status	Mut	144(56.2%)	97(38.5%)	<0.001
	WT	112(43.8%)	155(61.5%)	
KRAS status	Mut	74(28.9%)	65(25.8%)	0.492
	WT	182(71.1%)	187(74.2%)	

CR, complete response; PD, progressive disease; PR, partial response; SD, stable disease; Mut, mutation type; WT, wild type.

**Figure 2:**
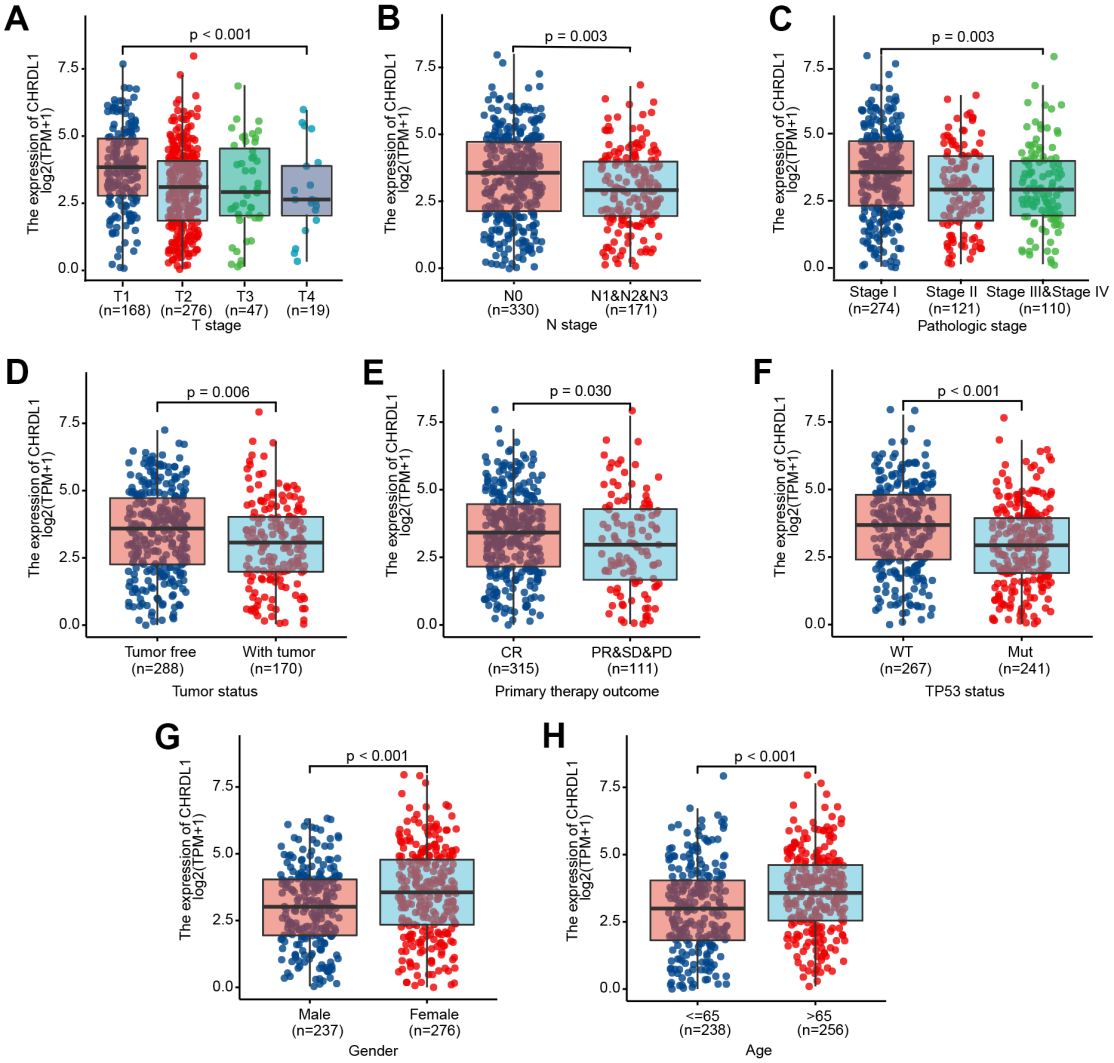
Association between CHRDL1 expression and clinicopathologic characteristics, including (**A**–**H**): T stage (P<0.001), N stage (P=0.003), Pathologic stage (P=0.003), Tumor status (P=0.006), Primary therapy outcome (P=0.030), TP53 status (P<0.001), Gender (P<0.001) and Age (P<0.001) in patients with LUAD in a TCGA cohort.

Then, 513 patients were separated into high and low CHRDL1 groups based on the median value of CHRDL1 expression. The results of the box plot showed that a lower level of CHRDL1 was significantly correlative with a higher T stage(*P*<0.001), N stage(*P*=0.019) and pathologic stage(*P*=0.007, Figure 2A–2C). The positive cancer status was correlated with lower CHRDL1 expression(*P*=0.006, Figure 2D). Moreover, poor treatment outcome(*P*=0.03) and TP53 mutation(*P*<0.001) were also significantly correlated with lower expression of CHRDL1 (Figure 2E, 2F). Finally, a higher level of CHRDL1 was significantly associated with female(*P*<0.001) and the elderly(age>65, *P*<0.001, Figure 2G, 2H).

Coincidentally, logistic regression indicated that lower CHRDL1 expression levels were significantly associated with poor prognostic characteristics (Table 2), including LUAD patients with a larger tumor extent (OR=0.45, *P*<0.001), higher level of regional lymph node invasion (OR=0.57, *P* <0.003), advanced pathologic stage (OR=0.53, *P* <0.001), worse treatment effect (OR=0.64, *P*=0.047), poor cancer status (OR=0.63, *P*=0.018), and TP53 mutation (OR=0.49, *P*<0.001). These results implied that LUAD patients with low CHRDL1 were more related to poor clinicopathological features.

**Table 2:** CHRDL1 expression associated with clinical pathological characteristics (logistic regression).

**Characteristics**	**Total number**	**OR (95%CI)**	***P* value**
T stage (T2&T3&T4 vs. T1)	510	0.45(0.30-0.65)	<0.001
N stage (N1&N2&N3 vs. N0)	501	0.57(0.39-0.83)	0.003
M stage (M1 vs. M0)	369	0.97(0.42-2.19)	0.936
Pathologic stage (Stage II&StageIII&Stage IV vs. Stage I)	505	0.53(0.37-0.75)	<0.001
Primary therapy outcome (PD&SD&PR vs. CR)	426	0.64(0.41-0.99)	0.047
Tumor status (With tumor vs. Tumor free)	458	0.63(0.43-0.92)	0.018
TP53 status (Mut vs. WT)	508	0.49(0.34-0.69)	<0.001
KRAS status (Mut vs. WT)	508	0.85(0.58-1.26)	0.432

OR, odds ratio; CI, confidence interval; CR, complete response; PD, progressive disease; PR, partial response; SD, stable disease; Mut, mutation type; WT, wild type.

### The Kaplan–Meier and Cox regression analysis of survival

**Figure 3:**
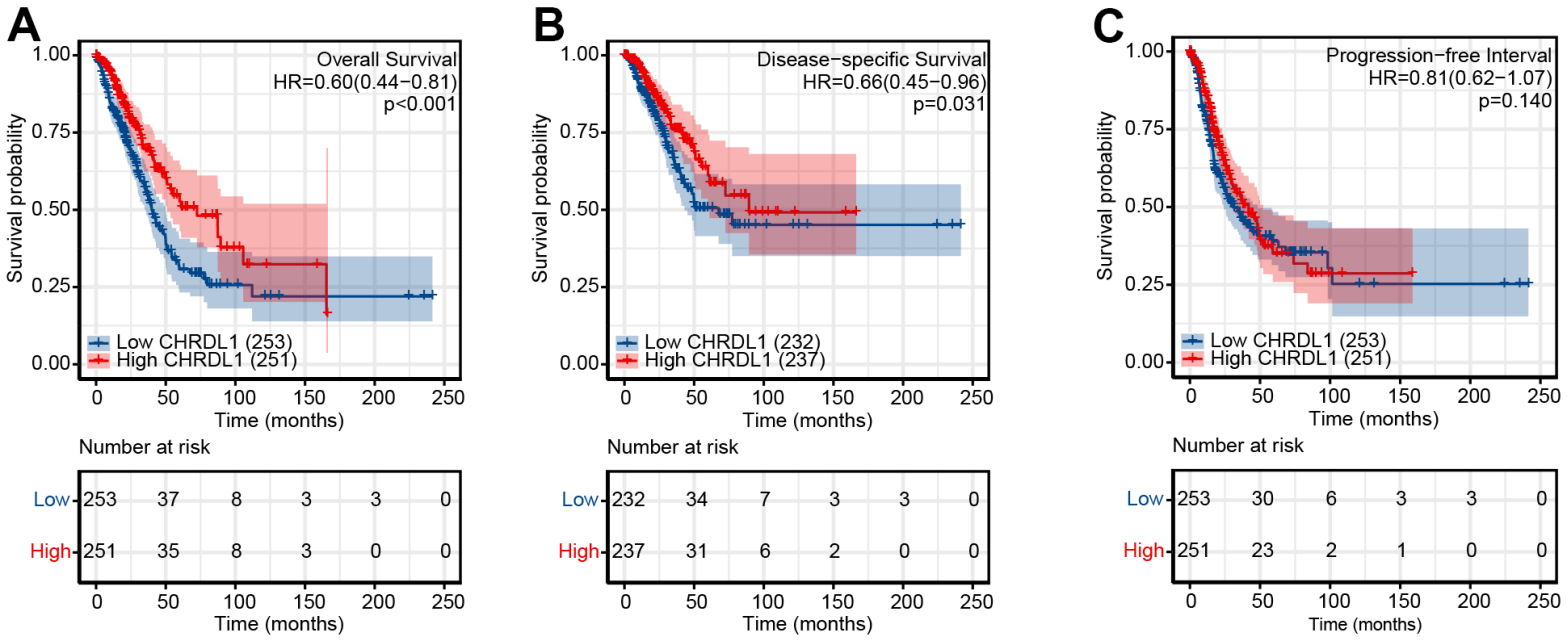
Impact of CHRDL1 expression on OS (**A**), DSS (**B**) and DFS (**C**) in patients with LUAD in a TCGA cohort. The bottom half of the picture showed that the risk table records the number of people who were still following at each point in time.

As shown in Figure 3, LUAD patients with low CHRDL1 had a worse prognosis than that with high CHRDL1. The Kaplan–Meier analysis illustrated that low CHRDL1 was significantly correlative with a poor OS (HR=0.60, *P*<0.001) and a shorter DSS (HR=0.66, *P*=0.031, Figure 3A, 3B). But there was no significant relevance between CHRDL1 expression and PFS (HR=0.81, *P*=0.14, Figure 3C). Univariate Cox regression analysis showed that several clinicopathologic characteristics were associated with poor OS (Table 3), including advanced T stage (HR=1.668, *P*=0.003), N stage (HR=2.606, *P*<0.001), M stage (HR=2.111, *P*=0.007), poor pathologic stage (HR=2.975, *P*<0.001), worse treatment effect (HR=2.818, *P*<0.001), positive tumor status (HR=6.211, *P*<0.001) and low CHRDL1 (HR=0.598, *P*<0.001). Furthermore, multivariate analysis suggested that CHRDL1 was independently correlative with OS (Table 3), with a HR of 0.563(*P*=0.016), along with primary therapy outcome (HR=2.022, *P*=0.002) and tumor status (HR=5.956, *P*<0.001).

**Table 3:** Associations between overall survival clinicopathological characteristics in TCGA patients with Cox regression.

**Characteristics**	**N**	**Univariate analysis**			**Multivariate analysis**	
		**HR(95% CI)**	***P* value**		**HR(95% CI)**	***P* value**
Gender (Female vs. Male)	504	0.943 (0.705-1.262)	0.694			
Age (>65 vs. <=65)	494	1.228 (0.915-1.649)	0.171			
Smoker (Yes vs. No)	490	0.887 (0.587-1.339)	0.568			
T stage (T2&T3&T4 vs. T1)	501	1.668 (1.184-2.349)	0.003		1.346 (0.789-2.296)	0.275
N stage (N1&N2&N3 vs. N0)	492	2.606 (1.939-3.503)	<0.001		1.542 (0.754-3.152)	0.235
M stage (M1 vs. M0)	360	2.111 (1.232-3.616)	0.007		0.864 (0.363-2.058)	0.741
Pathologic stage (Stage II&Stage III&Stage IV vs. Stage I)	496	2.975 (2.188-4.045)	<0.001		0.936 (0.425-2.059)	0.869
Primary therapy outcome (PR&SD&PD vs. CR)	419	2.818 (2.004-3.963)	<0.001		2.022 (1.284-3.184)	0.002
Tumor status (With tumor vs. Tumor free)	450	6.211 (4.258-9.059)	<0.001		5.956 (3.542-10.016)	<0.001
TP53 status (Mut vs. WT)	499	1.254 (0.936-1.680)	0.130			
KRAS status (Mut vs. WT)	499	1.087 (0.779-1.517)	0.623			
CHRDL1 (High vs. Low)	504	0.598 (0.444-0.807)	<0.001		0.563 (0.353-0.899)	0.016

HR, Hazard Ratio; CI, confidence interval; CR, complete response; PD, progressive disease; PR, partial response; SD, stable disease; Mut, mutation type; WT, wild type CR, complete response; PD, progressive disease; PR, partial response; SD, stable disease.

### A nomogram constructed by CHRDL1 and other independent clinical risk factors

**Figure 4:**
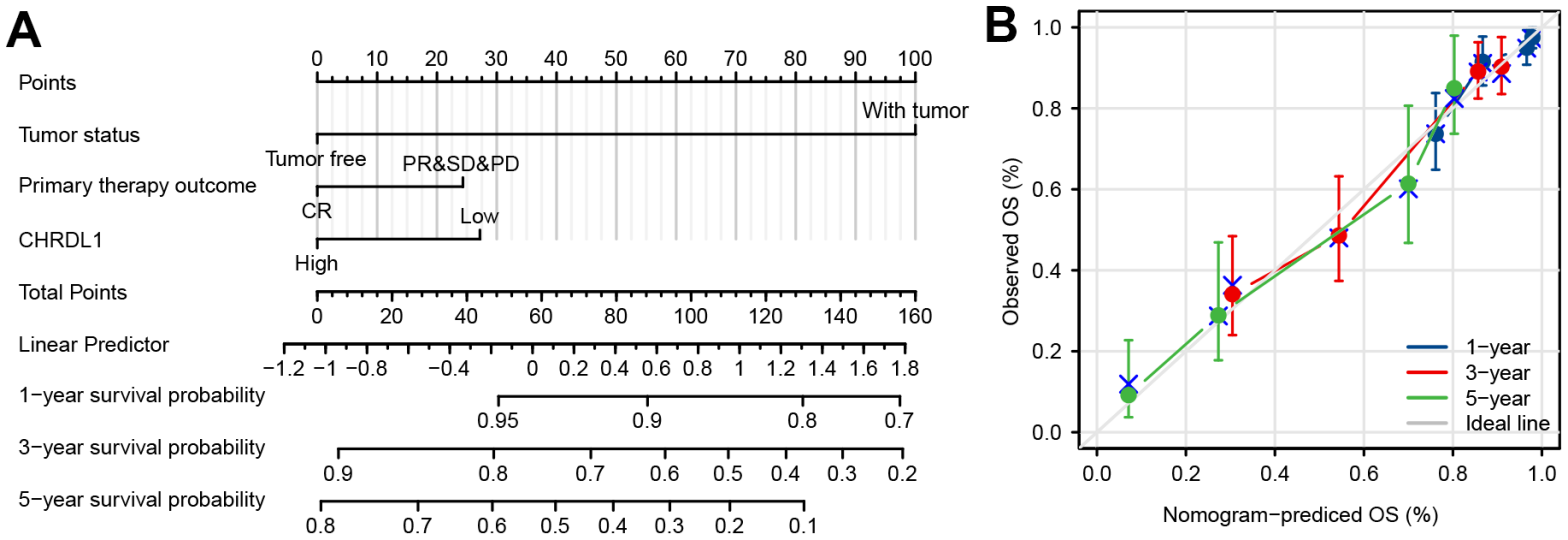
**Construction and validation of a nomogram based on the CHRDL1.** (**A**) nomogram to predict survival probability at 1, 3, and 5 years for LUAD patients. The C-index for the nomogram was 0.752(CI=0.727-0.776) with 1000 bootstrap replicates. (**B**) Calibration curve with the Hosmer-Lemeshow test of the nomogram in the TCGA-LUAD cohort.

To supply an available quantitative approach of predicting the prognosis to clinical worker for LUAD patients, a nomogram was established by CHRDL1 and other independent clinical risk factors (primary therapy outcome and tumor status). Obviously, the nomogram showed that CHRDL1 possessed a certain predictive efficacy for the prognosis of patients with LUAD (Figure 4A). And its C-index was 0.752(CI=0.727-0.776), which indicated that the predictive ability of the nomogram has medium accuracy. Besides, the calibration curve of the nomogram was found very close to the ideal 45° C curve, indicating favorable consistency between the prediction and the observation (Figure 4B). Thus, these results demonstrated that the nomogram was an effective measurement for predicting survival period in LUAD patients.

### Identification of DEGs

**Figure 5:**
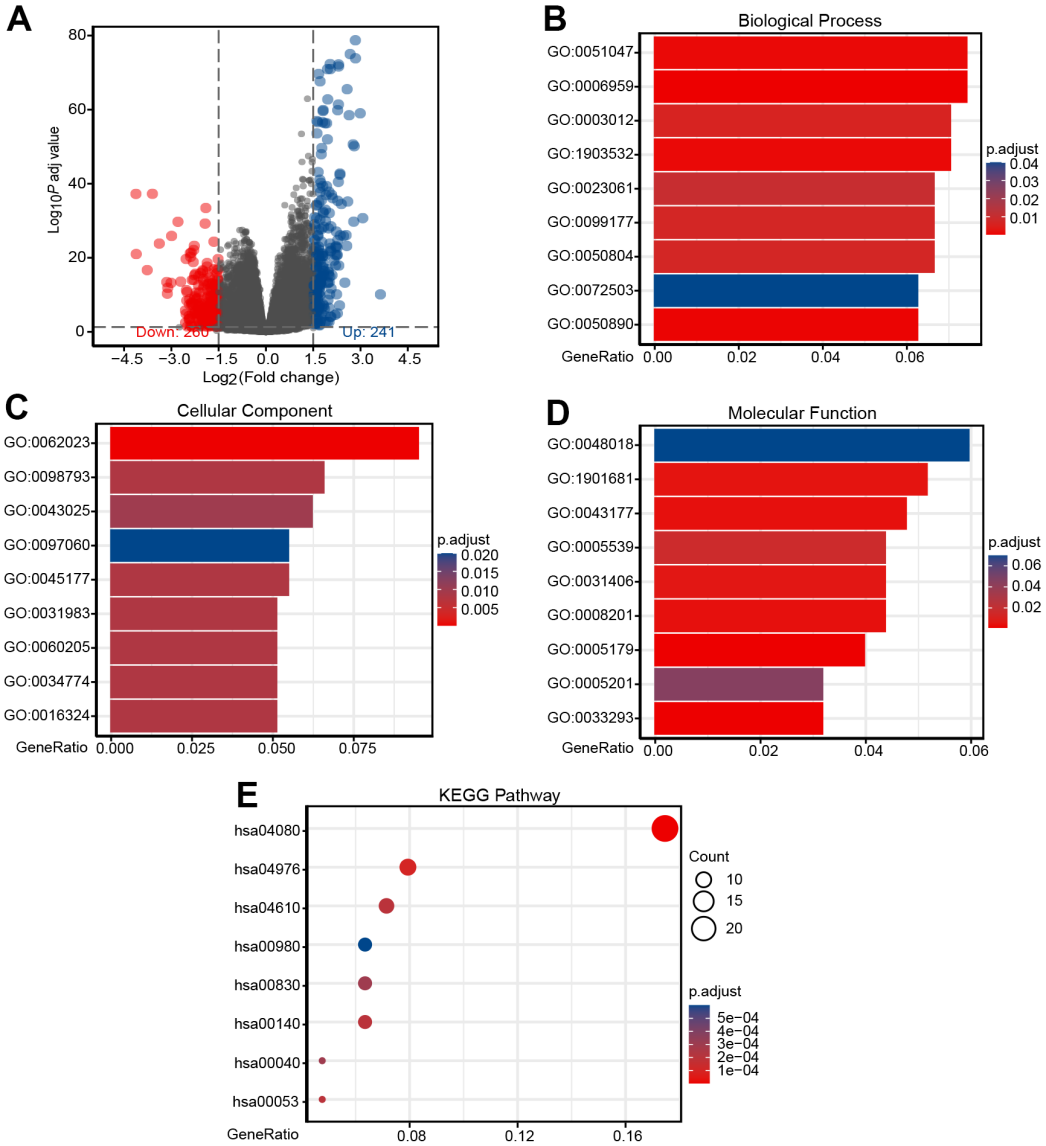
**Identification of DEGs and functional enrichment analysis of DEGs.** (**A**) Volcano plot of differential gene profiles between CHRDL1-high and -low groups. A total of 501 DEGs (241 upregulated and 260 downregulated) were identified. (**B**) Enriched GO terms in the "Biological Process" category. (**C**) Enriched GO terms in the "Cellular Component" category. (**D**) Enriched GO terms in the "Molecular Function" category. (**E**) Enriched GO terms in the "KEGG Pathway" category. The x-axis represents the proportion of DEGs, and the y-axis represents different categories. The colors indicate adjusted P-value, and the column lengths and circle sizes represent the enriched number of DEGs.

To better elucidate the underlying mechanisms of CHRDL1 expression in LUAD, a total of 501 DEGs were screened after the analyses of TCGA RNAseq data. DEGs expressions were illustrated by Volcano plot (Figure 5A). And PPI of CHRDL1 related co-expressed genes were established (Supplementary Figure 1).

### Functional enrichment analysis of DEGs

To further elucidate the role of CHRDL1 in LUAD, GO and KEGG analysis was performed by ClusterProfiler package (Table 4). In the biological process category [38], 225 enriched GO terms were discovered. Among them, they mainly participated in humoral immune response (GO:0006959), positive regulation of secretion (GO:0051047), positive regulation of secretion by cell (GO:1903532), muscle system process (GO:0003012), muscle system process modulation of chemical synaptic (GO:0050804) and regulation of trans-synaptic signaling (GO:0099177) (Figure 5B). Categorization by “cellular component” revealed 28 enriched terms, they were mainly enriched in collagen-containing extracellular matrix (GO:0062023), presynapse (GO:0098793), neuronal cell body (GO:0043025), apical part of cell (GO:0045177), synaptic membrane (GO:0097060) and apical plasma membrane (GO:0016324) (Figure 5C). In addition, the “molecular function” category disclosed 30 significant enrichment in terms and mainly associated with receptor ligand activity (GO:0048018), sulfur compound binding (GO:1901681), organic acid binding (GO:0043177), heparin binding (GO:0008201), carboxylic acid binding (GO:0031406) and hormone activity (GO:0005179) (Figure 5D). KEGG analysis revealed changes in genes sets related to neuroactive ligand-receptor interaction (hsa04080), complement and coagulation cascades (hsa04610), steroid hormone biosynthesis (hsa00140), retinol metabolism (hsa00830) and metabolism of xenobiotics by cytochrome P450 (hsa00980) [39, 40] (Figure 5E). These results may confirm the importance of CHRDL1 in body homeostasis and neural signal transduction.

**Table 4:** Functional enrichment analysis of DEGs.

**ONTOLOGY**	**ID**	**Description**	***P*.adjust**	**Count**
BP	GO:0006959	humoral immune response	1.412400E-04	19
BP	GO:0051047	positive regulation of secretion	1.009938E-03	19
BP	GO:1903532	positive regulation of secretion by cell	1.207653E-03	18
BP	GO:0003012	muscle system process	5.775649E-03	18
BP	GO:0050804	modulation of chemical synaptic transmission	6.625203E-03	17
BP	GO:0099177	regulation of trans-synaptic signaling	6.683871E-03	17
BP	GO:0023061	signal release	1.025742E-02	17
BP	GO:0050890	cognition	5.351450E-04	16
BP	GO:0072503	cellular divalent inorganic cation homeostasis	3.519247E-02	16
CC	GO:0062023	collagen-containing extracellular matrix	3.410000E-08	26
CC	GO:0098793	presynapse	7.581631E-03	18
CC	GO:0043025	neuronal cell body	1.252760E-02	17
CC	GO:0045177	apical part of cell	7.669858E-03	15
CC	GO:0097060	synaptic membrane	1.878734E-02	15
CC	GO:0016324	apical plasma membrane	7.581631E-03	14
CC	GO:0034774	secretory granule lumen	7.581631E-03	14
CC	GO:0060205	cytoplasmic vesicle lumen	7.669858E-03	14
CC	GO:0031983	vesicle lumen	7.669858E-03	14
MF	GO:0048018	receptor ligand activity	6.958732E-02	15
MF	GO:1901681	sulfur compound binding	4.876470E-03	13
MF	GO:0043177	organic acid binding	3.588468E-03	12
MF	GO:0008201	heparin binding	3.584050E-03	11
MF	GO:0031406	carboxylic acid binding	6.395279E-03	11
MF	GO:0005539	glycosaminoglycan binding	1.615000E-02	11
MF	GO:0005179	hormone activity	1.492445E-03	10
MF	GO:0033293	monocarboxylic acid binding	1.443176E-03	8
MF	GO:0005201	extracellular matrix structural constituent	4.511226E-02	8
	**ID**	**Description**	**P.adjust**	**Count**
KEGG	hsa04080	Neuroactive ligand-receptor interaction	2.590000E-06	22
KEGG	hsa04610	Complement and coagulation cascades	3.407000E-04	9
KEGG	hsa00140	Steroid hormone biosynthesis	2.986370E-04	8
KEGG	hsa00830	Retinol metabolism	3.666170E-04	8
KEGG	hsa00980	Metabolism of xenobiotics by cytochrome P450	7.403330E-04	8
KEGG	hsa05204	Chemical carcinogenesis	9.972090E-04	8
KEGG	hsa00053	Ascorbate and aldarate metabolism	2.986370E-04	6
KEGG	hsa00040	Pentose and glucuronate interconversions	4.249400E-04	6
KEGG	hsa00860	Porphyrin and chlorophyll metabolism	9.972090E-04	6

BP, biological process; CC, cellular component; MF, molecular function.

### Signaling pathways related CHRDL1 based on GSEA

**Figure 6:**
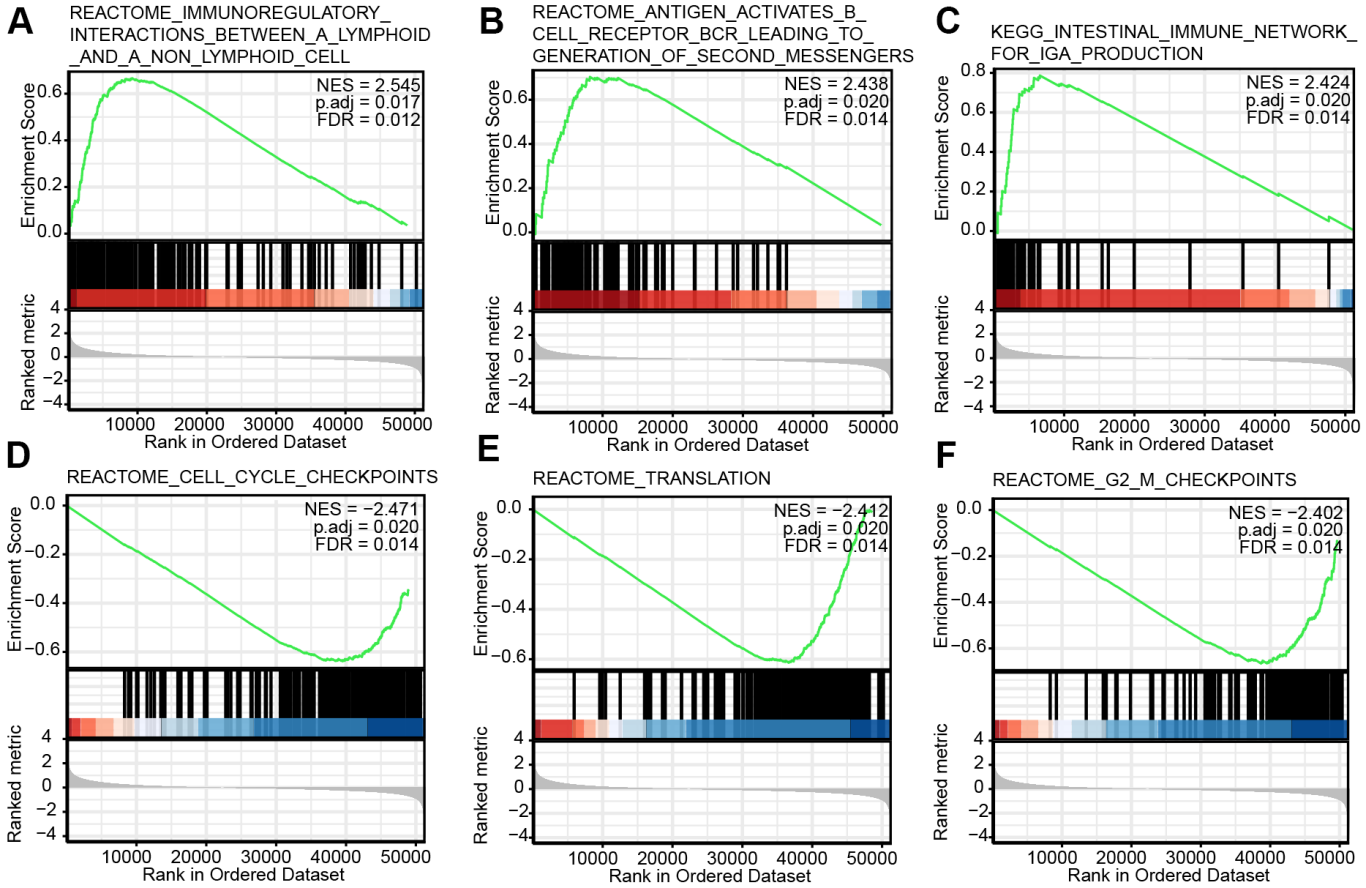
**Enrichment plots from the gene set enrichment analysis (GSEA).** (**A**–**F**) Several biological processes and pathways were differentially enriched in CHRDL1-related LUAD. NES, normalized ES; p.adj, adjusted P-value; FDR, false discovery rate.

Signaling pathways related CHRDL1 were identified by GSEA (Table 5). Three pathways showed significantly differential enrichments in CHRDL1 high expression phenotype (Figure 6A–6C), including immunoregulatory interactions between a lymphoid and a non-lymphoid cell, antigen activates B cell receptor (BCR) leading to generation of second messengers and intestinal immune network for IgA production. And cell cycle checkpoints, translation and G2M checkpoints suggested significantly differential enrichment in low CHRDL1 group (Figure 6D–6F). These results suggested the link between CHRDL1 and immune regulation and cell cycle in LUAD.

**Table 5:** CHRDL1 related signaling pathways based on GSEA.

**ID**	**Set size**	**Enrichment score**	**NES**	***P*.adjust**	**FDR**
REACTOME Immunoregulatory interactions between a lymphoid and a non lymphoid cell	179	0.666	2.564	0.02	0.014
REACTOME Antigen activates B cell receptor (BCR) leading to generation of second messengers	83	0.704	2.438	0.02	0.014
KEGG Intestinal immune network for IgA production	45	0.787	2.424	0.02	0.014
REACTOME G2M checkpoints	145	-0.666	-2.402	0.02	0.014
REACTOME Translation	287	-0.614	-2.412	0.02	0.014
REACTOME Cell cycle checkpoints	263	-0.638	-2.471	0.02	0.014

NES, normalized enrichment score; FDR, false discovery rate.

### The correlation between CHRDL1 and immune infiltration

**Figure 7:**
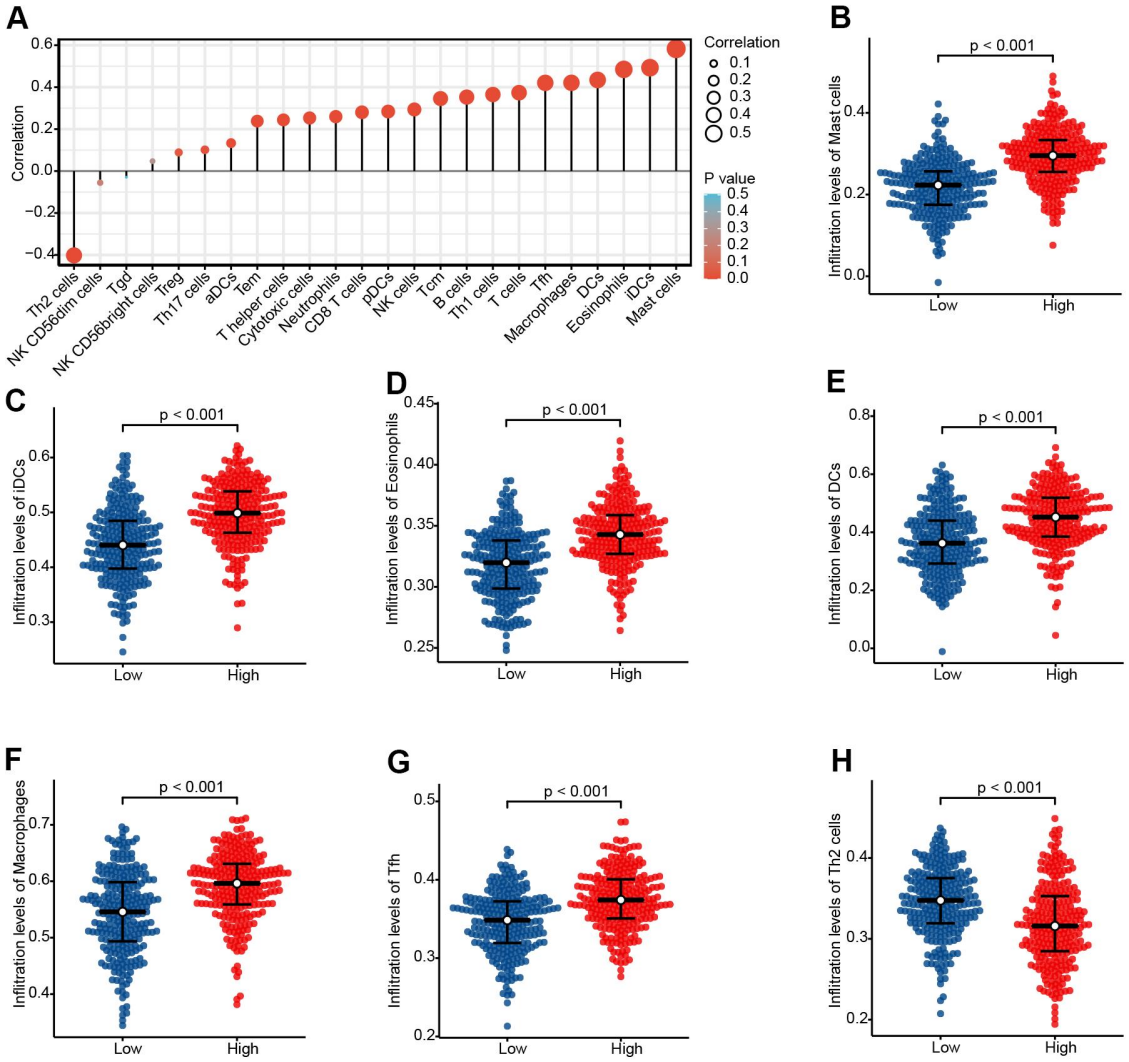
**The expression level of CHRDL1 was associated with the immune infiltration in the tumour microenvironment.** (**A**) Correlation between the relative abundances of 24 immune cells and CHRDL1 expression level. The size of the dots shows the absolute value of Spearman r. The colors represent the P-value. (**B**–**H**) The difference of immune cells infiltration level between CHRDL1 high and low expression groups was analyzed by Wilcoxon rank sum test.

Immune infiltration analysis showed that CHRDL1 was significantly associated with 7 kinds of immune cells (Figure 7A and Supplementary Figure 2), including Mast cells (R=0.583, *P*<0.001), iDCs (R=0.493, *P*<0.001), Eosinophils (R=0.485, *P*<0.001), DCs (R=0.434, *P*<0.001), Macrophages (R=0.421, *P*<0.001), Tfh (R=0.421, *P*<0.001) and Th2 cells (R=–0.402, *P*<0.001). Then, the differences of infiltration level for these immune cells between high and low CHRDL1 groups were analyzed (*P*<0.001, Figure 7B–7H).

### Experimental verification of the expression and prognosis value of CHRDL1 by IHC

**Figure 8:**
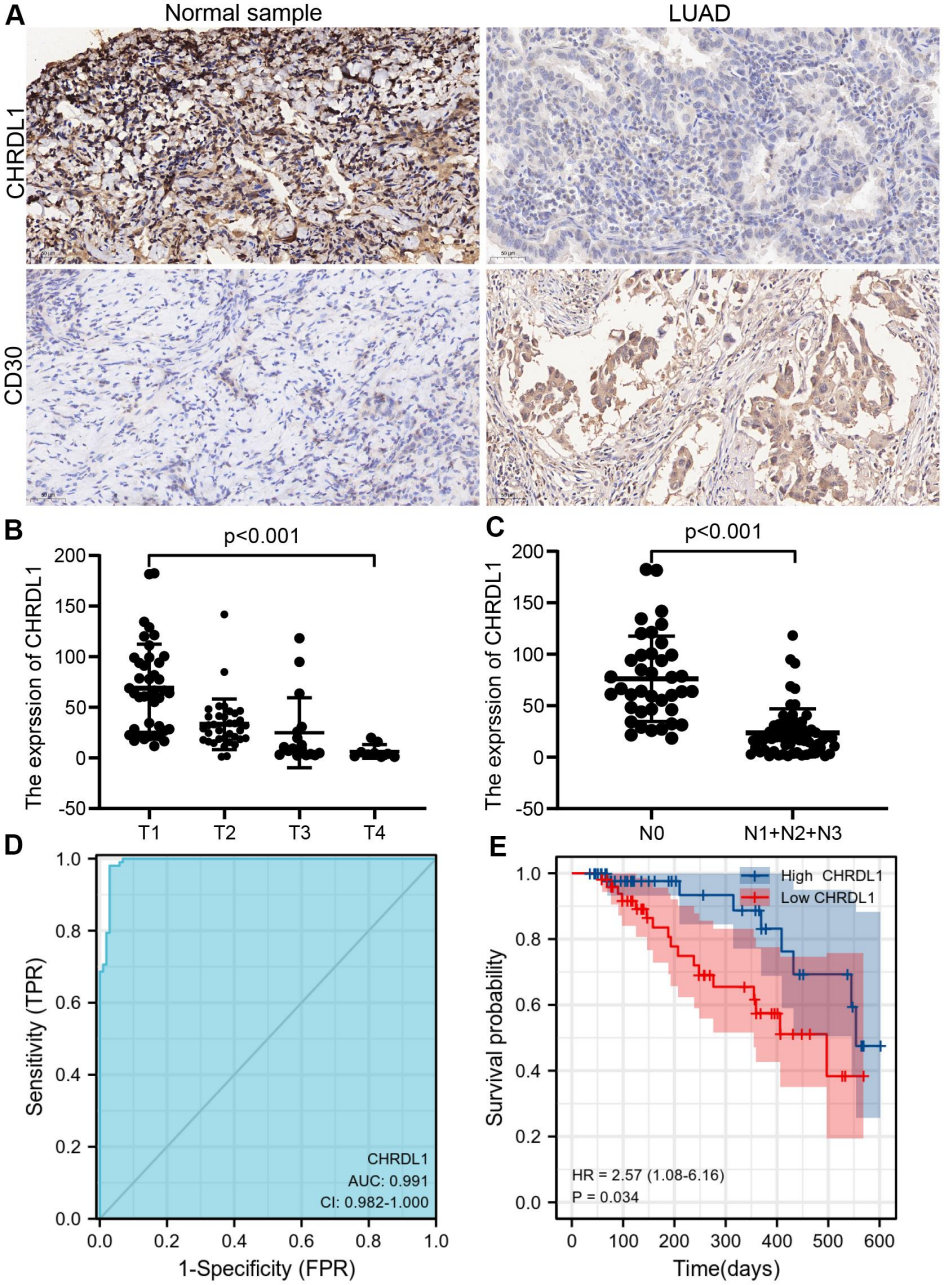
**The experimental validation of CHRDL1 by IHC.** (**A**) IHC staining of CHRDL1 and CD30 in normal sample and LUAD tissues. (**B**, **C**) Validation of association between CHRDL1 expression and TNM stage by IHC. (**D**) Validation of CHRDL1 expression showing discrimination power between normal samples and LUAD tissues by IHC. (**E**) Validation of the impact of CHRDL1 expression on overall survival in LUAD patients.

Immunohistochemistry (IHC) was applied to 102 LUAD tissues and 102 normal samples (Figure 8A). The results indicated that CHRDL1 expression was significantly elevated in LUAD tissues comparing to normal samples. To verify the negative relation between CHRDL1 and Th2 cells, CD30 was detected in LUAD tissues, which can reveal the activity of Th2 cells. Our results suggested that CD30 was significantly upregulated in normal samples, which has the opposite result to expression of CHRDL1 in normal samples. Moreover, CHRDL1 was quantified by integrated optical density (IOD) in 204 samples. Combined with the clinical information, the results verified that a lower level of CHRDL1 was significantly correlative with a higher T stage(*P*<0.001) and N stage (*P*<0.001, Figure 8B, 8C). ROC curve verified that CHRDL1 had a good ability to distinguish LUAD tissue from normal tissue (AUC=0.991,Figure 8D). Results of the survival curve illustrated that the patients with low CHRDL1 had poor OS(*P*<0.034, Figure 8E). These results confirmed the prognostic value including the correlation between CHRDL1 and patients TNM stage and survival time.

## DISCUSSION

CHRDL1 (Chordin-like1) belongs to the Chordin family and is located on the X chromosome [13, 41]. Its function is mainly as a specific inhibitor of bone morphogenetic proteins (BMPs), especially bone morphogenetic protein4 (BMP4) [18, 42–44]. The abnormal expression of BMP4 has been confirmed in various cancers, including NSCLC [45, 46]. Interestingly, the role of BMP4 in LUAD and LUSC is completely different. BMP4 could serve as a tumor suppressor in LUSC and inhibited the growth of LUSC cells [45]. In LUAD, BMP4 knockout could impede migration and invasion of LUAD cells [46]. However, CHRDL1, as a secretory antagonist of BMP4, is known little about its role in LUAD.

Recently, with the update of second-generation sequencing technology and establishment of public databases for sharing clinical data, more and more bioinformatics studies have achieved satisfactory results in the identification of key genes [47, 48]. In our study, RNA-sequencing profiles from TCGA were used to investigate the expression and prognosis value of CHRDL1 in LUAD. We found that CHRDL1 was significantly decreased in the majority of cancers. At present, the research of CHRDL1 in tumor is very scarce. In malignant melanoma, CHRDL1 was identified aa a tumour-suppressor gene and was significantly down-regulated in melanoma cell lines [16]. Abnormally elevated CHRDL1 was also found in T cell acute lymphoblastic leukemia [17]. Besides, down-regulation of CHRDL1 was observed in breast and gastric cancer [18, 19]. All the findings about the expression of CHRDL1 were consistent with our results in pan-cancer, and this also proved the reliability and authenticity of our study. Absolutely, the down-regulated expression of CHRDL1 in LUAD also was verified according to our experimental validation by IHC.

Besides, it seems that the clinical significance of CHRDL1 is very impressive in our clinical correlation analysis. Our study suggested that low CHRDL1 in LUAD was significantly related to advanced clinicopathological features(high T and N stage, positive tumor status, poor treatment effect and TP53 mutation). TNM stage has been used to assess the severity of tumor extent, lymph node invasion and distant metastasis since 1966 [49]. In our study, CHRDL1 is only related to T and N stage, not to M stage. This maybe indicate that CHRDL1 is mainly involved in the regulation of tumor growth rather than tumor migration and invasion. The therapeutic efficiency of anti-cancer therapy is a critical factor affecting the long-term survival of cancer patients [50]. This suggested that CHRDL1 could predict patients who will be more conductive to anti-cancer therapy. Logistic regression also verified that CHRDL1 is a protective factor for LUAD patients in the above aspects. Noticeably, high levels of CHRDL1 are also a protective factor against TP53 mutations. As we known, TP53 is famous for a tumor suppressor gene, which plays an essential role in the maintenance of cell proliferation and apoptosis homeostasis [51]. The mutation of TP53 is one of the most common genetic mutations in cancers [52]. This may imply that CHRDL1 could affect the proliferation of tumor cells through TP53 mutations, just as we mentioned above affecting the T stage. Simultaneously, univariate and multivariate regression analysis suggested that CHRDL1 is an independent prognostic factor. Furthermore, the OS period of the high CHRDL1 group was significantly prolonged. The above results illustrated that CHRDL1 maybe a prognostic biomarker and possess promising prognostic value for LUAD patients.

The promising prognostic value of CHRDL1 makes us yearn for latent molecular mechanisms and regulatory networks. To further elucidate the relevant underlying cellular mechanisms of CHRDL1 in LUAD, GO and KEGG analysis were performed in subsequent study. Certainly, the results were consistent with previous studies. It was mainly related to biological process and pathway of secretion and nerve synapse, as CHRDL1 is a secretory protein and involved in neurodevelopment [14, 15]. On the other hand, we also found that CHRDL1 was associated with maintenance of homeostasis and immune regulation, such as complement and coagulation system, body hormone synthesis and humoral immune response. In order for the above results to be confirmed more convincingly, GSEA analysis was performed. The results suggested that CHRDL1 was indeed related to the homeostasis of cell proliferation, such as cell cycle checkpoint, protein translation and G2M phase checkpoint. The cell cycle is almost the most vital pathway regulating proliferation of tumor cells [53]. Therefore, the abnormal decrease of CHRDL1 may cause the excessive proliferation of cancer cells through activation of cell cycle checkpoint. At present, cell cycle checkpoint inhibitors (ATR, CHK1 and WEE1) have been already an general and effective measure for anti-cancer therapy [54–56]. And the role of tumor suppressor TP53 in triggering cell cycle checkpoints has been confirmed [57]. The above findings indicated that CHRDL1 could serve as a potential therapy target on inhibiting the proliferation of LUAD cells by cell cycle checkpoint.

On the other hand, interestingly, we found that CHRDL1 has a certain relationship in in lymphocyte immunoregulatory interactions through GSEA analysis. The immunomodulatory function of CHRDL1 has not been reported yet. A recent clinical trial study found that tumour-infiltrating lymphocyte abundance is significantly correlative with promising outcome in patients with advanced NSCLC treated with immunotherapy [58]. This suggested that CHRDL1 may affect the efficiency of immunotherapy by regulating immune infiltration. Thus, we calculated the abundance correlation between 24 types of immune cells and CHRDL1 expression. Obviously, CHRDL1 expression significantly correlated with 7 kinds of immune cells. And most of them belong to innate immunity (mast cells, eosinophils, iDCs, DCs and macrophages). As we known, innate immunity is a natural barrier for human beings to foreign antigens and maintains the homeostasis [59–61]. Studies have shown that lack of innate immunity was an essential factor for the emergence and development of lung cancer [62]. This suggests that CHRDL1 may be able to protect our body against the invasion of cancer through innate immunity.

Lately, immunotherapy has emerged as a prospective treatment for [58]. But only a minority of cancer patients have benefited from immunotherapy successfully. Tumor-specific cytotoxic T lymphocytes (CTLs) have been regarded as a powerful method of antitumor immunotherapy [63, 64]. Th1 cells appear to be essential for the optimum generation and the durability of specific CTLs [65]. In fact, Th2 cells always significantly limit current immunotherapy through negatively regulating Th1-type immunity [66]. Therefore, our study found that Th2 cells were negatively correlated with CHRDL1, whereas Th1 cells and CHRDL1 were positive correlated. This also explains why patients with low CHRDL1 expression had poor treatment outcome. The former studies suggested that CD30 was preferably expressed on CD4+ T cells, which produce Th2 cytokines [67, 68]. Thus, we indirectly confirmed a negative correlation between CHRDL1 and Th2 cells by immunohistochemical detection of CD30. These findings indicated that CHRDL1 might improve the effectiveness of immunotherapy in LUAD by regulating immune infiltration.

Although our study served as a precedent for elucidating the expression and prognostic value of CHRDL1 in LUAD, there are still some limitations. First of all, our validation of prognostic value of CHRDL1 was imperfect due to insufficient sample size and time limitation. For example, the sample did not include enough patients with distant metastasis, and the follow-up time was not long enough. So more clinical trials are needed to complete more adequate validation of prognostic value of CHRDL1 in the future. Second, although we discussed the possible mechanism of CHRDL1 in regulating LUAD, there are some deviations to using transcriptome to predict protein expression [69]. Thus, more wet experiments are required to identify the specific biological functions and regulatory mechanisms of CHRDL1 in LUAD.

In conclusion, our study demonstrated the expression and prognostic value of CHRDL1 in LUAD. CHRDL1 may be a potential therapy target through cell cycle checkpoint and improve the effectiveness of immunotherapy by regulating immune infiltration. Our study supplies novel and prospective insights for subsequent research to clarify the clinicopathological importance and molecular pathogenesis of LUAD.

## Supplementary Material

Supplementary Figures
