# Supplementary material for: Chordin-like 1 is a novel prognostic biomarker and correlative with immune cell infiltration in lung adenocarcinoma
Source: Aging (Albany NY). 2022 Jan 12;14(1):389–409. doi: 10.18632/aging.203814 (PMC8791215; doi:10.18632/aging.203814)
Supplement: Supplementary Figures [file aging-14-203814-s001.pdf]

SUPPLEMENTARY FIGURES

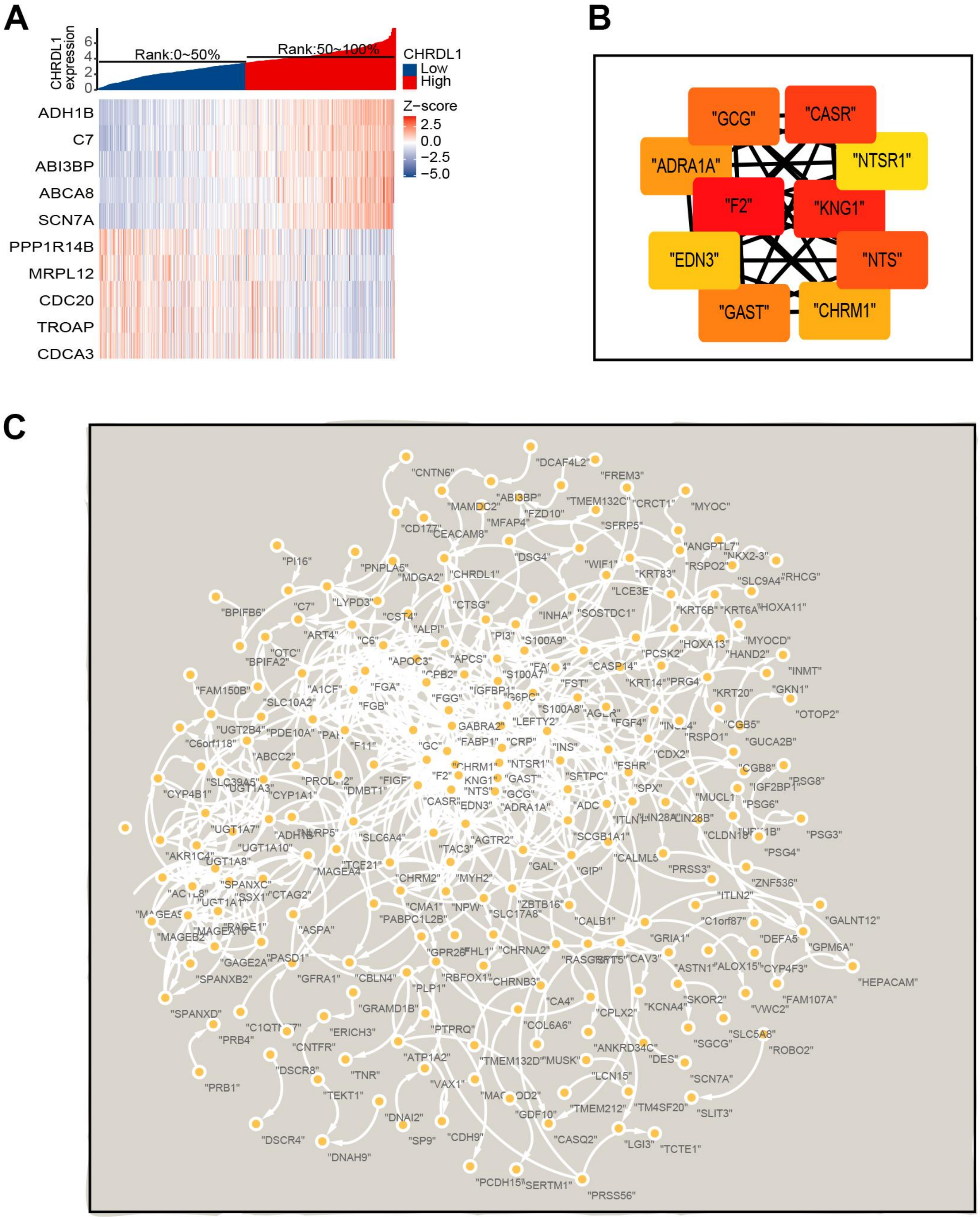

Supplementary Figure 1. Establishment of protein-protein interaction (PPI) network. (**A**) Heat map of CHRDL1 related co-expressed genes. (**B**) CHRDL1 related hub genes screened by Hubba plug-in algorithm of Cytoscape. (**C**) Establishment of protein-protein interaction (PPI) network based on CHRDL1.

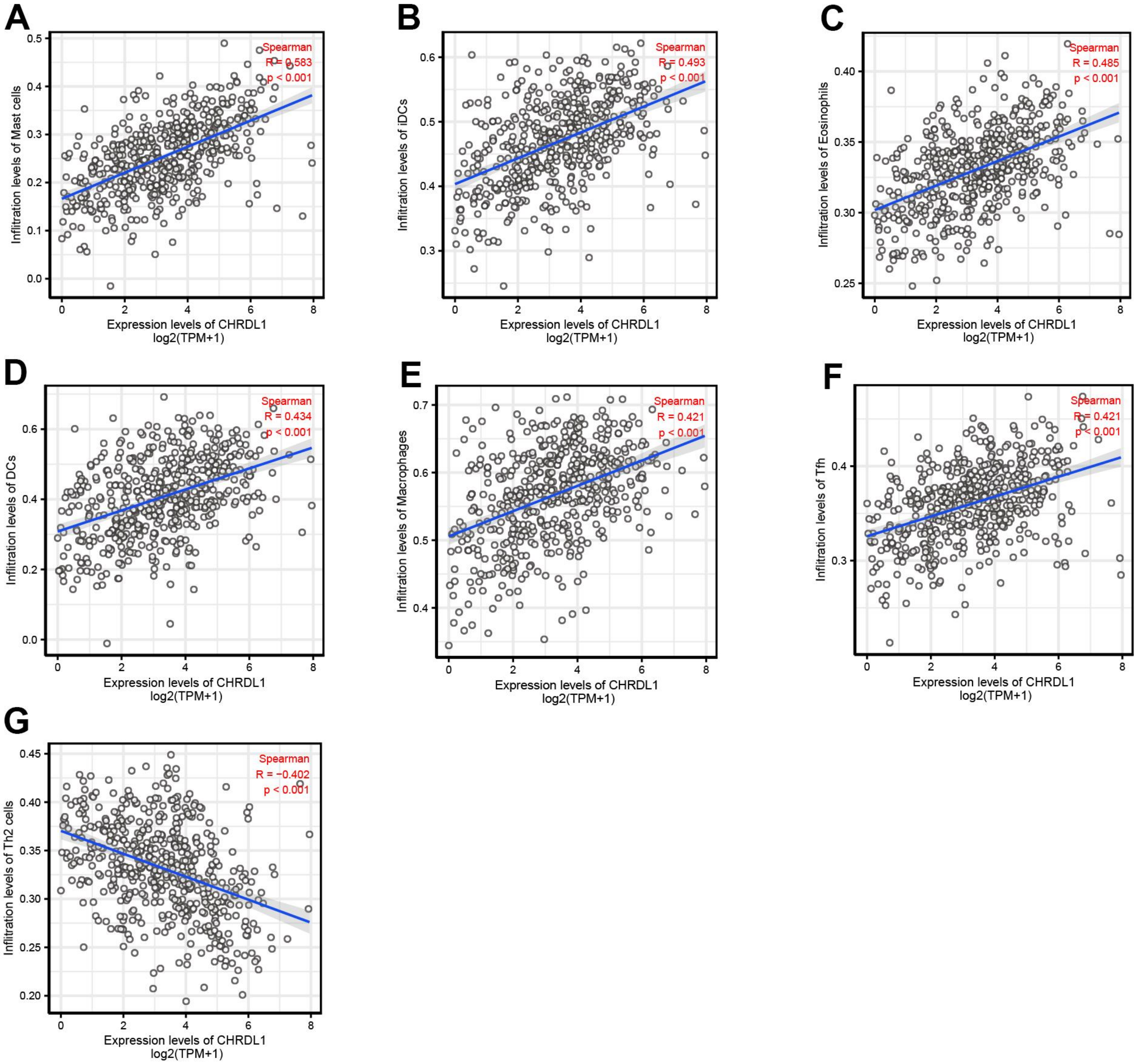

Supplementary Figure 2. The expression of CHRDL1 was significantly correlated with the infiltration level of 7 kinds of immune cells, including(**A**–**G**): Mast cells, iDCs, Eosinophils, DCs, Macrophages, Tfh and Th2 cells.
